# CD163^+^ tumor‐associated macrophage accumulation in breast cancer patients reflects both local differentiation signals and systemic skewing of monocytes

**DOI:** 10.1002/cti2.1108

**Published:** 2020-02-13

**Authors:** Rodrigo Nalio Ramos, Céline Rodriguez, Margaux Hubert, Maude Ardin, Isabelle Treilleux, Carola H Ries, Emilie Lavergne, Sylvie Chabaud, Amélie Colombe, Olivier Trédan, Henrique Gomes Guedes, Fábio Laginha, Wilfrid Richer, Eliane Piaggio, José Alexandre M Barbuto, Christophe Caux, Christine Ménétrier‐Caux, Nathalie Bendriss‐Vermare

**Affiliations:** ^1^ INSERM U1052 CNRS 5286 Centre Léon Bérard Centre de Recherche en Cancérologie de Lyon Univ Lyon Université Claude Bernard Lyon 1 Lyon France; ^2^ Department of Immunology Institute of Biomedical Sciences – University of São Paulo São Paulo Brazil; ^3^ Centre Léon Bérard Lyon France; ^4^ Roche Pharmaceutical Research and Early Development Roche Innovation Center Munich Penzberg Germany; ^5^ Perola Byington Hospital São Paulo Brazil; ^6^ Institut Curie PSL Research University Paris France; ^7^ INSERM U932 Paris France

**Keywords:** breast cancer, CD163, IFN responses, IL‐10, tumor‐associated macrophages

## Abstract

**Objectives:**

The accumulation of tumor‐associated macrophages (TAMs) is correlated with poor clinical outcome, but the mechanisms governing their differentiation from circulating monocytes remain unclear in humans.

**Methods:**

Using multicolor flow cytometry, we evaluated TAMs phenotype in 93 breast cancer (BC) patients. Furthermore, monocytes from healthy donors were cultured in the presence of supernatants from dilacerated primary tumors to investigate their differentiation into macrophages (MΦ) *in vitro*. Additionally, we used transcriptomic analysis to evaluate BC patients’ blood monocytes profiles.

**Results:**

We observed that high intra‐tumor CD163‐expressing TAM density is predictive of reduced survival in BC patients. *In vitro*, M‐CSF, TGF‐β and VEGF from primary tumor supernatants skewed the differentiation of healthy donor blood monocytes towards CD163^high^CD86^low^IL‐10^high^ M2‐like MΦ that strongly suppressed CD4^+^ T‐cell expansion *via* PD‐L1 and IL‐10. In addition, blood monocytes from about 40% of BC patients displayed an altered response to *in vitro* stimulation, being refractory to type‐1 MΦ (M1‐MΦ) differentiation and secreting higher amounts of immunosuppressive, metastatic‐related and angiogenic cytokines. Aside from showing that monocyte transcriptome is significantly altered by the presence of BC, we also demonstrated an overall metabolic de‐activation in refractory monocytes of BC patients. In contrast, monocytes from sensitive BC patients undergoing normal M1‐MΦ differentiation showed up‐regulation of IFN‐response genes and had no signs of metabolic alteration.

**Conclusion:**

Altogether, our results suggest that systemic factors skew BC patient blood monocytes towards a pro‐metastatic profile, resulting in the accumulation of further polarised CD163^high^ TAMs resembling type‐2 MΦ (M2‐MΦ) in the local BC microenvironment. These data indicate that monitoring circulating monocytes in BC patients may provide an indication of early systemic alterations induced by cancer and, thus, be instrumental in the development of improved personalised immunotherapeutic interventions.

## Graphical abstract

The mechanisms governing suppressive tumor‐associated macrophage (TAM) generation from circulating monocytes remain unclear in humans. In this study, we identify tumor‐derived M‐CSF, TGF‐β and VEGF as key factors skewing monocytes towards immunosuppressive CD163^high^CD86^low^IL‐10^high^ M2‐like macrophages (MΦ). We also describe an intrinsic metabolic de‐activation in breast cancer (BC) patient monocytes refractory to type‐1 MΦ (M1‐MΦ) differentiation that contrast with the active intrinsic IFN‐signalling pathway detected in sensitive BC patient monocytes undergoing normal M1‐MΦ differentiation. Thus, the combined local and systemic skewing of monocytes may give rise to the accumulation of suppressive M2‐like TAMs, contributing to the immune response failure and impacting on BC patient outcome.

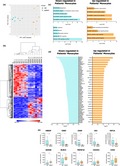

## Introduction

During cancer development, a complex microenvironment is formed, generating a unique set of signals impacting infiltrating immune cells. One consequence is the accumulation of tumor‐associated macrophages (TAMs), which are often abundantly present in malignant solid tumors and have been associated with tumor invasion, migration and angiogenesis,^1^, ^2^ as well as worse clinical outcome.^3^, ^4^

Tumor‐associated macrophages are mostly derived from circulating monocytes and can be classified by an oversimplified bi‐functional model of M1‐MΦ (inflammatory) versus M2‐MΦ (anti‐inflammatory) differentiation.^5^, ^6^ M1‐MΦ are recognised as classically activated MΦ endowed with anti‐tumoral properties, while M2‐MΦ contribute to tumor development because of their immunosuppressive and pro‐angiogenic features.^7^, ^8^ The use of large‐scale single‐cell analyses has recently revealed a new level of diversity in TAM populations according to their ontogeny and functional state that extends beyond the M1‐ and M2‐like phenotypes.^9^, ^10^, ^11^ However, the mechanisms and tumor‐derived factors responsible for educating monocytes to TAMs with different phenotypes by tumor‐derived factors remain poorly characterised in humans.

Importantly, several recent pre‐clinical and clinical data highlight that cancer progression is driven not only by genetic alterations in tumors and interactions with their local microenvironment, but also by complex and poorly understood systemic processes, which may have a profound impact on anti‐tumor immune responses.^12^ In this context, we have previously shown that circulating monocytes from breast cancer (BC) patients fail to differentiate into functional dendritic cells (DCs)^13^, ^14^ and present an altered cytokine profile in response to *in vitro* stimulation.^15^ Furthermore, other recent studies also highlighted the altered profile of circulating myeloid cells in both human^16^, ^17^, ^18^, ^19^ and mouse^20^, ^21^, ^22^ cancer‐bearing hosts, strongly suggesting a systemic role for tumors in skewing monocytes.

Using biological and transcriptomic approaches, we report here that systemic factors skew the blood monocytes of BC patients towards an anti‐inflammatory/pro‐metastatic profile, which, in the local microenvironment, are further differentiated into immunosuppressive CD163^high^ M2‐like TAMs. Understanding the mechanisms by which tumor‐derived factors influence TAM phenotype, either in circulation or within the tumor milieu, can be critical for the development of novel anti‐tumor therapeutic approaches.

## Results

### Accumulation of CD163^+^ TAMs is associated with poor survival in BC patients

**Figure 1:**
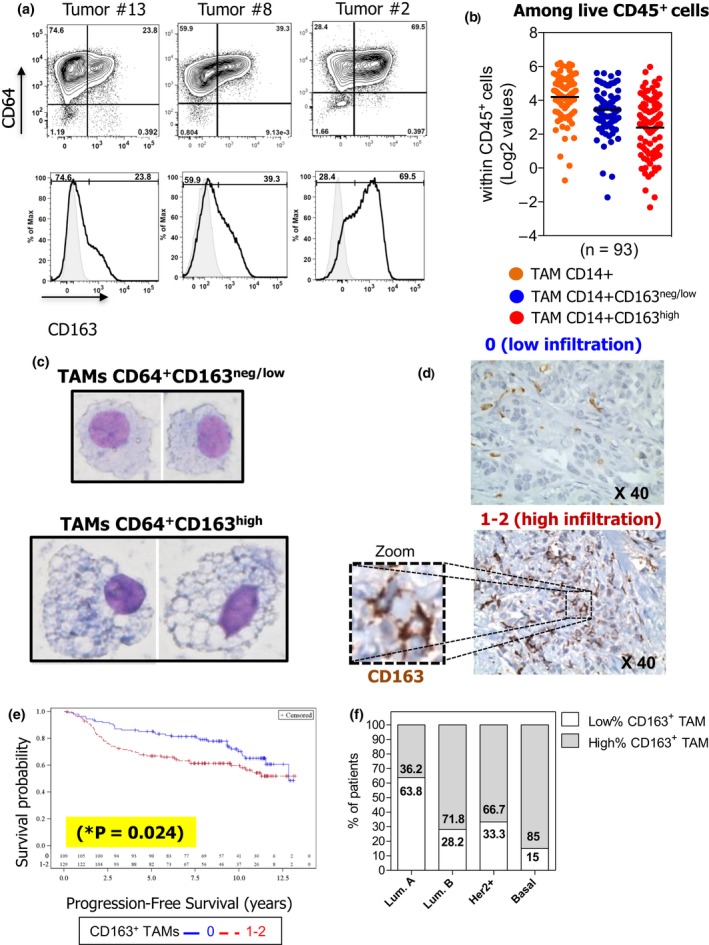
High frequency of CD163^+^ TAMs is correlated with higher risk of relapse in BC patients. CD163^neg/low^ and CD163^high^ TAMs within live CD45^+^CD11b^+^HLA‐DR^+^CD14^+^CD64^+^ cells from BC suspensions were analysed by FACS **(a)** (control isotype in grey). Dot plots shown are representative of each TAM profile for CD163 expression (low, intermediate and high) among 93 BC patients analysed. **(b)** Presence of total CD14^+^ TAMs, CD14^+^CD163^neg/low^, and CD14^+^CD163^high^ TAMs subsets among total live leucocytes (*n* = 93; horizontal bars represent the mean). Values depicted were log(base2)‐transformed from percentages obtained by FACS. **(c)** One representative May–Grünwald–Giemsa staining for sorted CD163^neg/low^ and CD163^high^ TAMs (objective 40x) obtained for one BC patient out of two performed. **(d)** Different levels of CD163^+^ TAM infiltration detected in TMAs: 0, low infiltration and 1–2, high infiltration. **(e)** Analysis of the PFS of the 238 BC patients according to their high (red line, *n* = 129) or low (blue line, *n* = 109) level of CD163^+^ TAM infiltration. **(f)** Frequency of CD163^+^ TAMs in patients according to their BC molecular subtype.

Tumor‐associated macrophage infiltrates in primary BC were characterised by multicolour flow cytometry, using the gating strategy shown in Supplementary figure 1a. TAMs were identified as CD45^+^CD11b^+^HLA‐DR^+^CD14^+^BDCA1^neg^CD64^+^ cells and formed two distinct clusters, namely CD163^neg/low^ and CD163^high^, presenting variable patterns among patients (Figure 1a). A similar profile was obtained by analysing a large cohort of BC patients (Supplementary table 1, *n* = 93). Total CD14^+^ TAMs represented about 25% (± 17.6%) of living CD45^+^ cells, with CD163^neg/low^ and CD163^high^ TAMs representing 15% (± 11.6%) and 9.7% (± 11%), respectively (data are presented as log_2_ values, Figure 1b). FACS‐sorted TAM subpopulations displayed distinct morphologies, CD163^high^ TAMs being larger and more vacuolated than CD163^neg/low^ TAMs (Figure 1c).

We analysed the impact of TAMs on patient survival by IHC evaluation of the frequency of CD163^+^ TAMs on paraffin‐embedded TMA from a retrospective cohort of 238 primary BC patients. According to pathologist evaluation, tumors were classified as presenting ‘low’ (0) or ‘high’ (1–2) CD163^+^ TAM infiltration (Figure 1d). Univariate analysis revealed that high CD163^+^ TAM infiltration was correlated with more aggressive tumors (high SBR grade, lymph node involvement and lymphatic emboli; Supplementary table 2) and was associated with poor progression‐free survival (PFS) compared to patients with low‐CD163 TAM expression (Figure 1e; *log‐rank *P*‐value = 0.024). Importantly, the negative impact of high CD163^+^ TAM infiltration on the PFS was still observed when the analysis was focused on non‐triple negative BC (TNBC) patients (*n* = 207 patients; log‐rank *P*‐value = 0.0022). This result demonstrates that the negative impact of high CD163^+^ TAM infiltration on PFS (Figure 1e) is not exclusively attributable to the higher infiltration of TNBC by CD163^+^ TAMs (Figure 1f).

### TGF‐β, M‐CSF and VEGF derived from the tumor microenvironment educate monocytes into suppressive CD163^high^CD86^low^IL‐10^high^ MΦ

**Figure 2:**
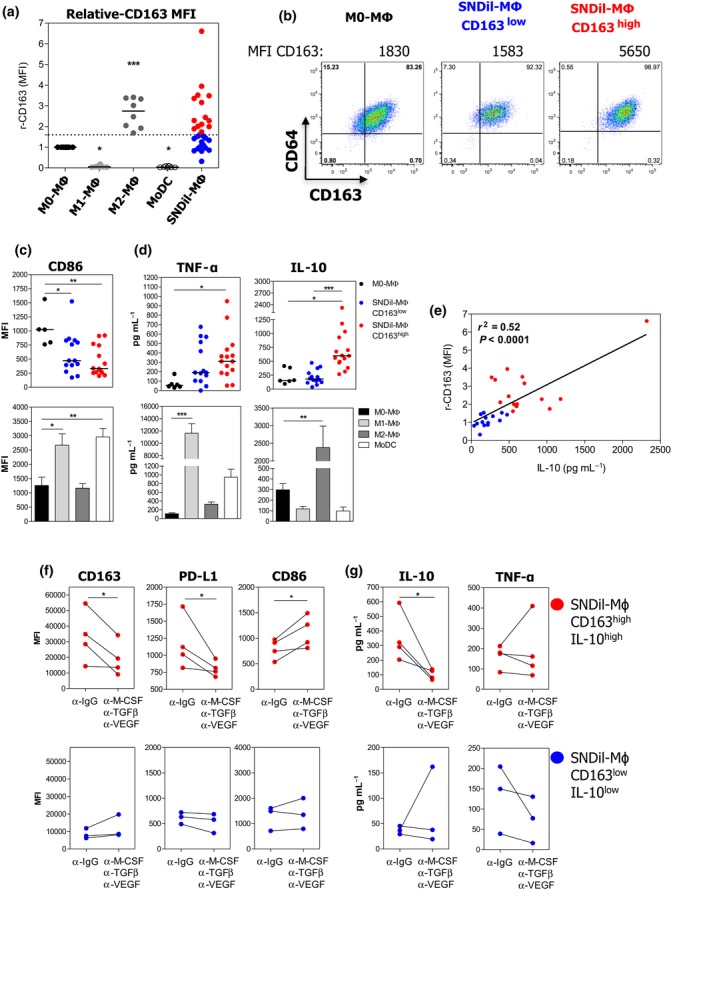
Tumor environmental factors turn monocytes into CD163^high^CD86^low^IL‐10^high^ MΦ. HD CD14^+^ monocytes were cultured in the presence of 25% SNDils for 7 days, and surface markers and cytokine production were evaluated 24 h after addition of LPS. **(a)** r‐CD163 MFI from control APCs (*n* = 8 independent donors) and SNDil‐MΦ (*n* = 29 independent SNDils). **(b)** Representative pseudocolour plots of CD64^+^CD163^+^ cells of M0‐MΦ and SNDil‐MΦ based on the median obtained within each group. **(c)** Expression of CD86 and **(d)** production of TNF‐α and IL‐10 in CD163^low^ and CD163^high^ SNDil‐MΦ or in control APCs. For **(a–d)**, experiments performed with, at least, five independent donor monocytes and different SNDils (*n* = 29). **(e)** Correlation of r‐CD163 MFI and IL‐10 production among SNDil‐MΦ. **(f, g)** SNDil‐MΦ were cultured in the presence of neutralising anti‐M‐CSF, anti‐TGF‐β and anti‐VEGF or control antibodies during the differentiation process and were activated by LPS during the last 24 h to evaluate surface markers **(f)** and cytokine production **(g)** in CD163^high^IL‐10^high^ SNDil‐MΦ (red dots, *n* = 4; *P ≤ 0.05) and CD163^low^IL‐10^low^ SNDil‐MΦ (blue dots; *n* = 3).

To decipher the impact of the tumor microenvironment on TAM populations, we investigated whether soluble factors present in primary tumor supernatants could affect the differentiation/function of healthy donor (HD) blood monocytes. CD14^+^ monocytes purified from HD blood were cultured for 7 days in the presence of SNDils (supernatant from primary dilacerated tumors) and analysed after 24 h of LPS activation for the expression of surface molecules and cytokine production. Results were compared to those observed in control M0‐MΦ, M1‐MΦ, M2‐MΦ and Mo‐DCs differentiated *in vitro* under well‐defined conditions. All MΦ populations (M0‐MΦ, M1‐MΦ, M2‐MΦ and SNDil‐MΦ) were characterised as CD14^+^CD64^+^BDCA1^low^, and Mo‐DCs as CD14^low^CD64^neg^BDCA‐1^high^. Among the cells differentiated under controlled conditions, M2‐MΦ displayed the highest levels of r‐CD163, while CD163 was lost in M1‐MΦ and Mo‐DCs (Supplementary figure 1b; Figure 2a). Interestingly, we found heterogeneous levels of r‐CD163 in SNDil‐MΦ, indicating a tumor‐dependent phenomenon (Figure 2a and b). A CD163^high^ phenotype (Figure 2a, red dots), similar to M2‐MΦ, was obtained in 51% (15/29) of SNDil‐MΦ, whereas the other SNDils (14/29) induced a CD163^neg/low^ phenotype (Figure 2a, blue dots), mostly resembling M0‐MΦ.

Regardless of r‐CD163 levels, SNDil‐MΦ showed significantly lower CD86 levels than M0‐MΦ (Figure 2c). However, CD163^high^ SNDil‐MΦ produced significantly higher IL‐10 levels (but not TNF‐α) than CD163^neg/low^ SNDil‐MΦ (Figure 2d). Of note, CD86 and TNF‐α were statistically higher in M1‐MΦ (Figure 2c and d), while a significant up‐regulation of IL‐10 was observed in M2‐MΦ (Figure 2d). Interestingly, we also found a positive correlation between r‐CD163 and IL‐10 levels considering all SNDil‐MΦ (Figure 2e). Moreover, the addition of neutralising anti‐IL‐10Rα mAb to M2‐MΦ cultures demonstrated that autocrine IL‐10 production is critical for the up‐regulation of CD163 (mean MFI ± SEM: anti‐IgG: 4538 ± 779 vs anti‐IL‐10Rα: 790 ± 127, *n* = 3) and PD‐L1 (mean MFI ± SEM anti‐IgG: 3626 ± 361 vs anti‐IL‐10Rα: 1755 ± 410, *n* = 3), but did not significantly alter PD‐L2, CD80 or CD86 expression (Supplementary figure 2a).

To identify SNDil tumor microenvironmental factors responsible for M2‐like MΦ differentiation, we quantified 48 cytokines and chemokines in SNDils by conducting a multiplexed immunoassay. Elevated amounts of CCL2, M‐CSF, TGF‐β1, TGF‐β3 and VEGF were detected in SNDils promoting CD163^high^IL‐10^high^ MΦs compared to those promoting CD163^low^IL‐10^low^ MΦ (Supplementary figure 2b). The addition of specific Abs neutralising M‐CSF, pan TGF‐β and VEGF during differentiation significantly impaired CD163, PD‐L1 and IL‐10 induction and led to a CD86 increase in SNDils promoting CD163^high^IL‐10^high^ MΦ, whereas no modulation was observed for those inducing CD163^low^IL‐10^low^ MΦ (Figure 2f and g).

**Figure 3:**
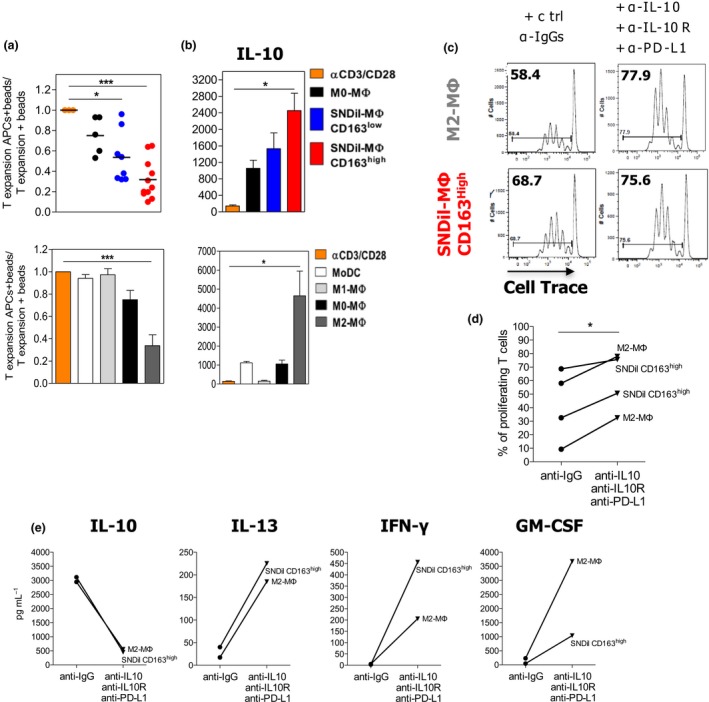
CD163^high^CD86^low^IL‐10^high^ SNDil‐MΦ suppress T‐cell proliferation. SNDil‐MΦ and control APCs were co‐cultured for 4 days with allogeneic naïve CD4^+^ T cells pre‐activated using expand beads. Evaluation of **(a)** T‐cell proliferation and **(b)** IL‐10 secretion in the co‐culture supernatants (SNDil‐MΦ CD163^low^ (blue, *n* = 8); SNDil‐MΦ CD163^high^ (red, *n* = 11); control APCs (*n* ≥ 5) (mean ± SEM; *P ≤ 0.05, **P ≤ 0.01; ***P ≤ 0.0001 to M0‐MΦ). Impact of neutralising anti‐IL‐10/IL‐10R and anti‐PD‐L1 antibodies on **(c, d)** T‐cell proliferation and **(e)** cytokine production in the same experimental settings as Figure 3a (*P ≤ 0.05). One representative experiment is shown in **c** and **e** out of two performed. **d** shows cumulative data of two independent experiments.

To investigate the functional consequences of tumor supernatants in MΦ differentiation, we evaluated the impact of differentiated MΦ on T‐cell proliferation and suppression. CD163^high^ SNDil‐MΦ strongly suppressed CD4^+^ T‐cell proliferation in response to anti‐CD3/anti‐CD28 beads compared to CD163^neg/low^ SNDil‐MΦ and M0‐MΦ (Figure 3a, upper panel) and were as potent as M2‐MΦ (Figure 3a, lower panel). Furthermore, significant up‐regulation of IL‐10 was observed only in co‐cultures containing CD163^high^ SNDil‐MΦ and M2‐MΦ (Figure 3b). Mechanistically, the simultaneous blockade of IL‐10/IL‐10Rα and PD‐L1 partly neutralised the suppressive functions of CD163^high^ SNDil‐MΦ and M2‐MΦ (Figure 3c and d), and partially restored IFN‐γ, GM‐CSF and IL‐13 production by activated‐CD4^+^ T cells (Figure 3e). Overall, these findings suggest that primary BC‐derived M‐CSF, VEGF and TGF‐β triggered IL‐10 production by monocytes, driving their final differentiation into immunosuppressive CD163^high^IL‐10^high^PD‐L1^+^CD86^low^ MΦ. Of note, we did not detect any specific pattern in SNDil from TNBC compared to other BC tumor subtypes.

### Blood monocytes from BC patients categorised as sensitive or refractory to GM‐CSF/IFN‐γ M1 differentiation

**Figure 4:**
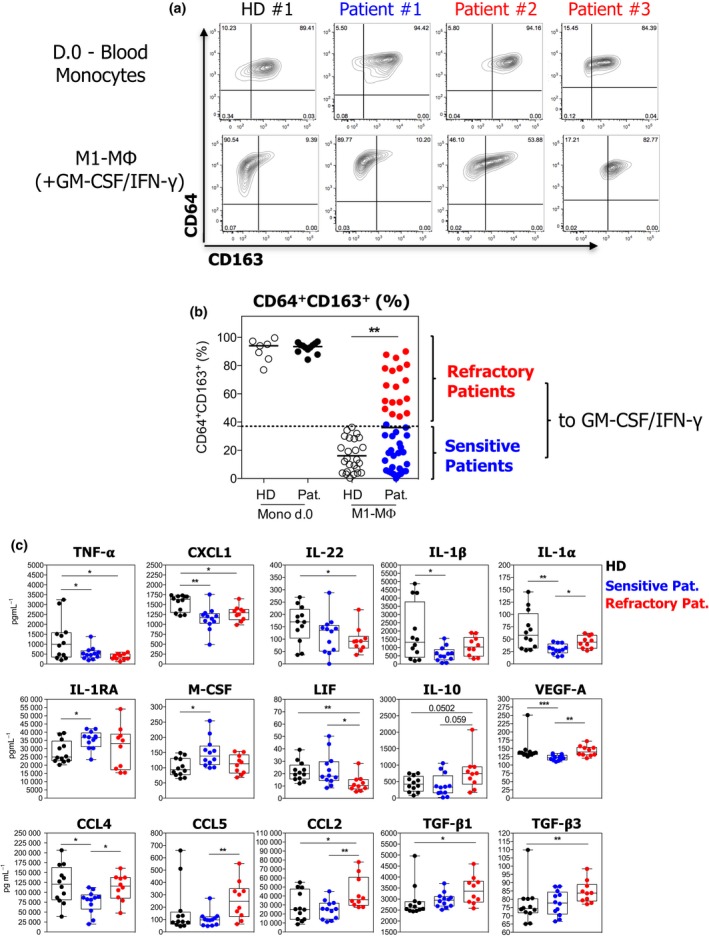
Blood monocytes from BC patients can be categorised as sensitive and refractory to GM‐CSF/IFN‐γ and produce distinct levels of cytokines upon stimulation. **(a, b)** BC patient blood monocytes were analysed for CD64 and CD163 expression at day 0 (at least *n* = 7) and after culture in M1‐MΦ condition (HD, *n* = 25; BC patients, *n* = 44; **P ≤ 0.01). **a** shows one representative dot plot of CD64 vs CD163 expression for each group. **(c)** BC patients and HD CD14^+^ monocytes were stimulated by LPS for 24 h. Levels of cytokines produced in monocyte supernatants from HDs (black, *n* = 12) and BC patients (blue: sensitive patients, *n* = 12; red: refractory patients, *n* = 10) are represented (*P ≤ 0.05, **P ≤ 0.01, ***P ≤ 0.0001).

Having demonstrated that the tumor microenvironment can drive the differentiation of monocytes into suppressive MΦ *in vitro*, we next investigated whether it could influence patient blood monocytes at distance via the bloodstream. To achieve this, fresh CD14^+^ blood monocytes from BC patients were cultured in the presence of a GM‐CSF + IFN‐γ (M1‐MΦ) cocktail. Although no difference was observed in CD163 levels at the beginning of the culture (Figure 4a and b, D0‐blood monocytes), 41% (18/44) of BC patient monocytes were refractory to M1‐MΦ differentiation since they did not down‐regulate CD163 expression under GM‐CSF/IFN‐γ culture as opposed to HD monocytes (*n* = 25; 36.1 ± 4.2% all patients vs 16 ± 2.3% HDs; Figure 4a and b). We also assessed the production of cytokines/chemokines of freshly isolated CD14^+^ patient monocytes under LPS stimulation (Figure 4c). Both patient monocyte subgroups produced significantly lower levels of TNF‐α and CXCL1 than HD monocytes. Refractory BC patient monocytes produced higher levels of IL‐10, CCL2, TGF‐β1 and TGF‐β3, but less IL‐22 and LIF than HD monocytes. Interestingly, refractory BC patient monocytes secreted elevated amounts of CCL2, CCL4, CCL5, IL‐1α and VEGF, and lower levels of LIF when compared to sensitive patient monocytes. Furthermore, considering all soluble factors, linear discriminant analysis (LDA) revealed that BC patient monocyte subgroups (refractory and sensitive to GM‐CSF) could be separated into 2 clusters by a combination of twelve cytokines (Supplementary figure 3).

### Tumor modulates transcriptional programming of BC patient monocytes

**Figure 5:**
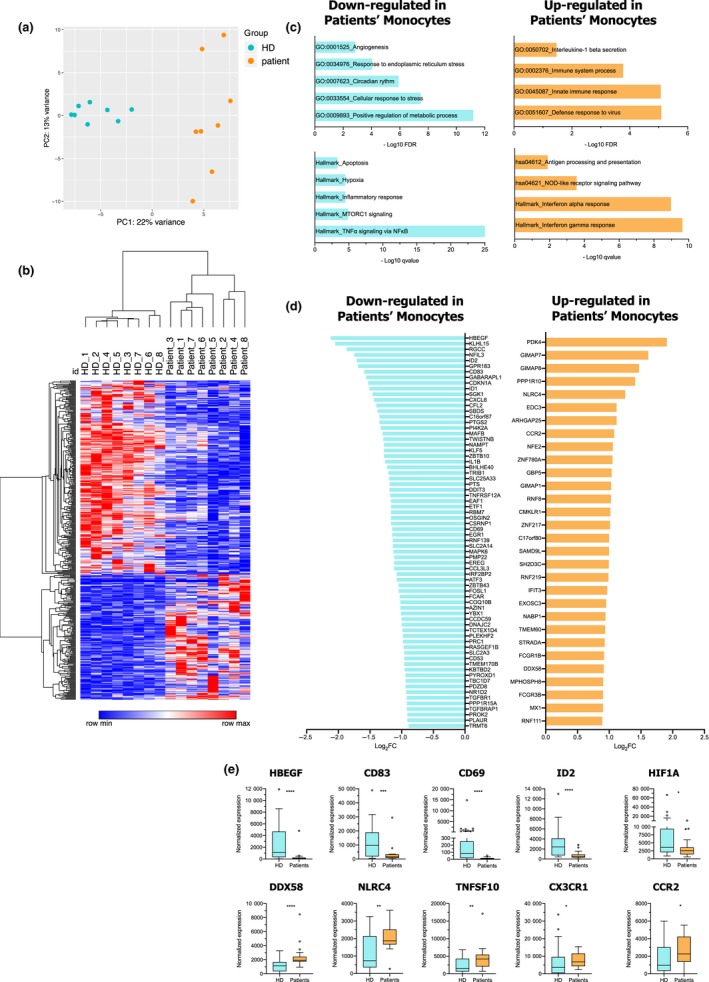
Transcriptomic profiles of blood monocytes from BC patients differ from HD monocytes. FACS‐sorted CD14^+^ blood monocytes from BC patients (*n* = 8) and HDs (*n* = 8) were submitted to transcriptome analysis. **(a)** Principal component analysis (PCA) plot from HD and patient monocytes considering the top 500 most variant genes. **(b)** Hierarchical clustering of all differentially expressed genes (DEGs) between BC patients and HD monocytes. **(c)** Pathway enrichment analysis (hallmark sets and Gene Ontology) of DEGs between BC patients and HD monocytes. **(d)** Bar plot of the top 100 DEGs between HD and patient monocytes (log_2_ fold change ≤ 0.58 and FDR ≤ 0.05). **(e)** Normalised expression of selected genes from our study in an independent cohort of BC from Cassetta *et al.* 18

To further investigate the systemic effect of the tumor in modulating patient monocytes, we evaluated the transcriptional profile of HD monocytes and both GM‐CSF refractory and sensitive patient monocytes through microarray analysis. Interestingly, principal component analysis (PCA) (Figure 5a) segregated patient monocytes from HD monocytes. We next applied a filter by considering genes with a log_2_ fold change ≥ 0.58 and FDR ≤ 0.05 comparing patient versus HD monocytes, and obtained 421 differentially expressed genes (DEGs) (Supplementary table 3). An unsupervised hierarchical clustering of these 421 DEGs confirmed the transcriptome alteration of BC patient monocytes compared to HD monocytes (Figure 5b). Running pathway enrichment analysis (MSigDB) (Figure 5c and Supplementary table 3) revealed that the most significant variations were hallmark of TNF‐alpha signalling *via* NF‐κB, down‐regulated in patient monocytes and hallmarks of both interferon alpha and gamma responses up‐regulated in patient monocytes. Additionally, Gene Ontology (GO) analysis revealed down‐regulation of metabolic processes, but up‐regulation in defence response to virus in patient monocytes, which is consistent with an up‐regulation of the IFN‐α response. Among the top 100 DEGs (Figure 5d and Supplementary table 3), 70 were down‐regulated in BC patient monocytes, including immune‐related genes already known to be involved in MΦ/DC differentiation (e.g. *MAFB* and *ID2*)^23^, ^24^ and immunity stimulation (e.g. *MAPK6* *, TNFRSF12A, CD69, PTGS2, FCAR, CD53, CD83* and *CXCL8*).^25^, ^26^, ^27^ Thirty genes were up‐regulated in BC patient monocytes, comprising genes encoding GTPases of the immunity‐associated protein (e.g. *GIMAP7* and *GIMAP8*), and genes involved in inflammasome signalling (e.g. *NLRC4*).^28^ Importantly, using recently published transcriptomic data sets of blood monocytes from an independent cohort of BC,^18^ we confirmed that genes such as *HBEGF*, *CD83*, *CD69*, *ID2* and *HIF1A* were statistically down‐regulated, while *DDX58*, *NLRC4*, *TNFSF10*, *CXC3R1* and *CCR2* genes were statistically up‐regulated in patient monocytes compared to HD monocytes (Figure 5e). Altogether, these findings revealed important differences in the transcriptional profiles of BC patient and HD monocytes, strongly suggesting that tumor development can act systemically, modifying the transcriptional profile of circulating monocytes.

**Figure 6:**
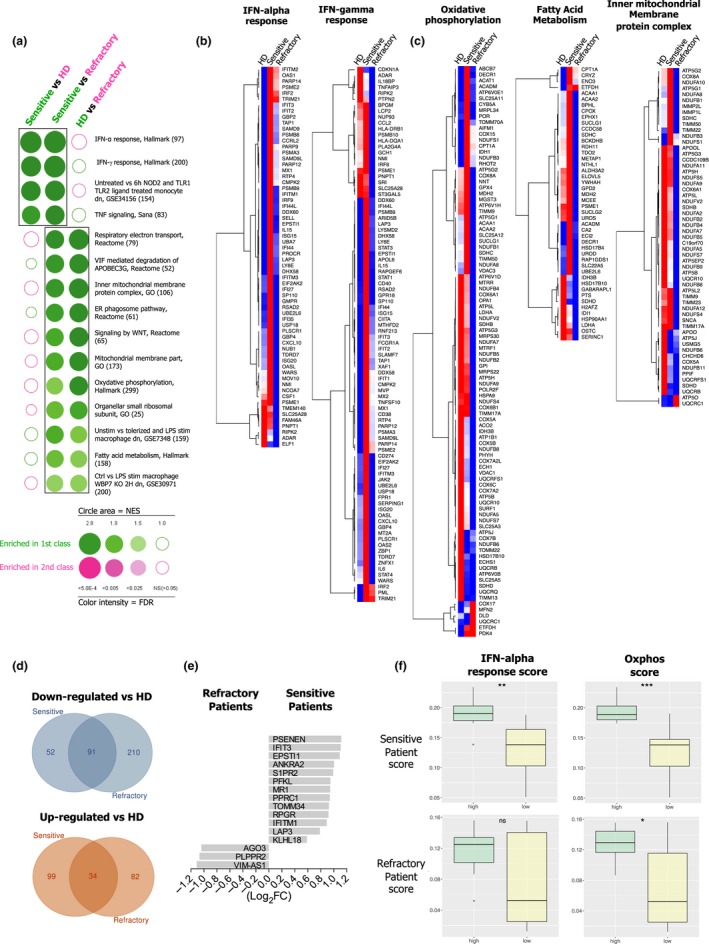
Monocytes from BC patients sensitive to M1 differentiation process display up‐regulated IFN‐signalling pathways. FACS‐sorted CD14^+^ blood monocytes from BC patients (sensitive, *n* = 4; refractory, *n* = 4) and HDs (*n* = 8) were submitted to transcriptome analysis. **(a)** BubbleGUM analysis for enriched sets of genes (GSEA) in sensitive, refractory and HD monocytes. Representative heat‐maps of selected sets of genes associated with **(b)** IFN‐α and IFN‐γ responses, and **(c)** oxidative phosphorylation, fatty acid metabolism and inner mitochondria membrane protein complex. **(d)** Venn diagram of commonly down‐regulated (blue circles) or up‐regulated (red circles) genes in monocytes from sensitive or refractory BC patients versus HDs. **(e)** Representation of 16 DEGs between sensitive and refractory BC patient monocytes. **(f)** Single‐sample scoring analysis of the sensitive and refractory signatures in BC patient cohorts from Cassetta *et al.* 18 stratified according to their type I IFN response or oxidative phosphorylation score.

We next performed GSEA stratifying patient monocytes according to their refractory and sensitive status to GM‐CSF/IFN‐γ responses. Interestingly, sensitive patient monocytes presented an enrichment in sets of genes associated with hallmarks of both IFN‐α and IFN‐γ responses when compared to refractory or HD monocytes (Figure 6a). This observation was thus attributed to the up‐regulation of genes including *XAF1, RASD2*, *MX1*, *IFIT2, IFIT3*, *IFI44*, *DDX58, IFITM1, IFITM3* and *ISG15* in sensitive patient monocytes (Figure 6b). Conversely, refractory patient monocytes displayed a reduced expression of the majority of gene sets tested, including metabolic‐related gene sets, namely oxidative phosphorylation, fatty acid metabolism and inner mitochondrial membrane protein complex (Figure 6a and c). Furthermore, sensitive and refractory patient monocytes displayed distinct DEG modulations with exclusively down‐regulated ones (sensitive = 52 genes,refractory = 210 genes) and up‐regulated ones (sensitive = 99 genes,refractory = 82 genes), compared to HD monocytes (Figure 6d and Supplementary table 4). In addition, analysis of DEGs between refractory and sensitive patient monocytes revealed only 16 genes (13 up‐regulated in sensitive, 3 up‐regulated in refractory; Figure 6e and Supplementary table 4). Importantly, we extracted gene signatures of refractory and sensitive patients (Supplementary table 5) using the GeneSign tool^25^ with the min–max method and a minimal log fold change (LFC) threshold to 1.2. We performed single‐sample scoring analysis to evaluate the sensitive and refractory signatures, as well as the hallmark signatures for IFN‐α response and OXPHOS identified in Figure 6a. We demonstrated in an independent cohort of BC patients^18^ that, unlike the refractory signature, the sensitive signature score was significantly higher (*P* = 0.0037) in monocytes with a high IFN signature score (Figure 6f). This sensitive signature score was also more significantly enriched in monocytes expressing a high OXPHOS score, compared to the refractory signature score, which was at the limit of significance when comparing monocytes with high versus low OXPHOS status in the Cassetta cohort (*P* = 0.045; Figure 6f).

Altogether, these results suggest that patient monocytes that normally respond to GM‐CSF/IFN‐γ cytokines exhibit an intrinsic activation of an IFN‐signalling set of genes that could allow patient stratification. Notwithstanding, refractory patient monocytes display non‐functional features evidenced by a down‐regulation in pathways associated with metabolisms.

## Discussion

We herein describe an important mechanism by which the tumor microenvironment educates human blood monocytes, providing a possible explanation for the generation/accumulation of suppressive TAMs in human BC. We identified soluble factors derived from the primary tumor microenvironment, responsible for the differentiation of suppressive CD163^high^CD86^low^IL‐10^high^ MΦ. Additionally, we show that blood CD14^+^ monocytes from BC patients are transcriptionally distinct from HD monocytes and could be categorised as sensitive or refractory to M1‐MΦ differentiation, under GM‐CSF/IFN‐γ. The transcriptional characterisation of these two BC patient monocyte subgroups revealed an intrinsic metabolic de‐activation in refractory monocytes that contrasted with the active intrinsic IFN‐signalling pathway detected in sensitive monocytes, thus allowing them to overcome the systemic negative tumor influence. Hence, we propose that this skewed differentiation capacity of BC patient monocytes, induced by the combined local and systemic skewing of tumor education, gives rise to suppressive M2‐like TAMs, contributing to the immune response failure and impacting BC patient outcome.

We identified about 25% of total immune cells infiltrating BCs as CD14^+^ TAMs that express variable levels of CD163, underlying the phenotypic heterogeneity in TAMs. Through high‐dimensional analysis approaches, several recent reports have uncovered TAM heterogeneity at the single‐cell level in distinct tumor types, including BC,^9^, ^29^ clear cell renal cell carcinoma,^10^ melanoma^30^ and lung adenocarcinoma.^11^, ^31^ These studies revealed a great variety of distinct phenotypic TAM subsets that co‐exist by sharing the expression of well‐established M1 and M2 markers. In agreement with our present work, these emerging studies suggest that TAMs may adapt to a variety of tumor microenvironmental clues during tumor development, by acquiring a large spectrum of states.^32^ Importantly, recent studies have shown that the use of immune checkpoint inhibitors such as anti‐PD‐1 and anti‐CTLA‐4 can modify the myeloid high‐dimensional landscape in mouse models,^33^, ^34^ providing new insights into its mechanisms of action and its clinical applications.

In addition, our IHC analysis revealed a positive association between high infiltration by CD163^+^ TAMs and poor prognosis, in accordance with other studies in BC.^35^, ^36^, ^37^, ^38^, ^39^ Additionally, recent reports have focused on CD163 status in specific well‐defined BC subtypes and under different therapies. In particular, CD163^+^ TAMs were shown in two independent cohorts of TNBC patients, to be associated with worse patient prognosis after adjuvant chemotherapy.^40^ Moreover, BCs harbouring the c‐Myb transcription factor known to be associated with a good prognosis^41^ display a reduced macrophage infiltration and express lower levels of CD163 mRNA.^42^ This may be because of the fact that c‐Myb regulates the transcriptional programme of tumor cells, reducing classical cytokines involved in monocyte recruitment and differentiation and angiogenesis such as *Ccl2*, *Csf2*, *Csf3*, *Vegfa* and *Vegfc*. INOS^+^ M1‐like TAMs, in contrast to CD163^+^ M2‐like TAMs, were associated with improved survival of trastuzumab‐treated HER2 amplified metastatic BC patients.^43^ Other very recent studies have demonstrated interesting strategies for the *in vivo* modulation of TAMs. In a murine colon adenocarcinoma model, treatment with two mAbs to concomitantly block CSF‐1R and stimulate CD40 resulted in the reprogramming of TAMs by increasing pro‐inflammatory signals such as IL‐12B, IL‐27, IL‐1β and CCL5.^44^ In addition, in a melanoma tumor model, the depletion of CD163^+^ TAMs, using an anti‐CD163 antibody conjugated to a doxorubicin charged liposome, led to a monocyte influx, the up‐regulation of IFN‐related cytokines and an anti‐tumor T‐cell response.^45^

Importantly, we described for the first time that conditioned‐media supernatants of dilacerated primary human breast tumors promote either highly suppressive CD163^high^IL‐10^high^CD86^low^ MΦ, resembling suppressive TAMs,^46^, ^47^, ^48^ or CD163^low^IL‐10^low^CD86^low^MΦ, displaying moderate suppressive functions. Aside from its central role in T‐cell suppression, we further documented the contribution of autocrine IL‐10 in the phenotypic/functional switch of monocytes into suppressive CD163^high^ MΦ, with low IL‐12p40 production and high PD‐L1 expression, in accordance with previous reports.^7^, ^49^ Indeed, both murine and human TAMs have been described to favor tumor progression through the promotion of angiogenesis^50^, ^51^ and the inhibition of T‐cell responses through IL‐10 and TGF‐β^49^, ^52^ or through the expression of inhibitory receptors, such as PD‐L1.^7^, ^53^ However, the association of immunosuppressive properties of TAMs with the CD163 status was poorly explored in humans.

We demonstrate for the first time that the combination of M‐CSF, TGF‐β and VEGF, all present in the BC primary tumor microenvironment, converts monocytes into suppressive CD163^high^CD86^low^IL‐10^high^ MΦ, whereas GM‐CSF and IFN‐γ, master M1‐MΦ inducers, were not detected in SNDils. This observation is concordant with (1) our pioneer works on M‐CSF and IL‐6 skewing monocytes into suppressive MΦ‐like cells, and blocking Mo‐DC differentiation^54^, ^55^ and (2) the ability of TGF‐β to enhance the suppressive phenotype of monocytes^56^, ^57^, ^58^ and pDC.^59^ Of importance, blocking M‐CSF, TGF‐β and VEGF also impairs IL‐10 production by SNDil‐MΦ. Although tumor‐derived factors have been extensively studied using cancer cell lines, our study is unique in demonstrating that primary BC environment‐derived M‐CSF, VEGF and TGF‐β promote IL‐10 production by monocytes, driving their final differentiation into immunosuppressive CD163^high^IL‐10^high^CD86^low^ MΦ. Importantly, other tumor microenvironment‐derived factors may also play a role in the TAM polarisation/differentiation. In particular, it was shown that breast tumor cell lines with epithelial‐to‐mesenchymal transition (EMT) features expressed AXL and that AXL/Gas6 signalling in TNBC patients can modulate TAM phenotype by inducing a M2‐like phenotype.^40^ In addition, cancer‐associated fibroblasts producing CXCL12 stimulate the migration of TAMs towards tumor‐associated vessels that may promote the extravasation of tumor cells to the bloodstream.^58^ In phyllode malignant breast tumors, a particular BC subtype, CCL5 produced by tumor cells promotes the polarisation of TAMs into IL18^+^ producers, *via* the AKT pathway leading to a positive feedback for tumor promotion and aggressiveness.^60^ Moreover under hypoxia, murine melanoma tumors secrete exosomes able to up‐regulate oxidative phosphorylation in bone marrow‐derived macrophages, inducing their polarisation towards F4/80^+^CD206^high^ M2‐MΦ, favoring tumor development.^61^

Outstandingly, our data suggest that tumor‐derived factors affect monocytes in the bloodstream of patient. Microarray analysis revealed that patient monocytes display a distinct transcriptome by down‐regulating genes involved in monocyte differentiation and immune stimulation, while producing fewer inflammatory cytokines than HD monocytes and these results have been confirmed in an independent set of transcriptomic data from patients with primary BC.^18^ These findings are in accordance with our previous studies, showing that blood monocytes from primary BC give rise to Mo‐DCs that favor Treg responses, partially *via* TGF‐β.^13^, ^14^ Furthermore, this monocyte skewing phenomenon depends on the tumor burden, since a functional recovery of Mo‐DCs is observed after tumor resection^62^ or after immunotherapeutic DC vaccination.^63^ Most interestingly, we highlight a heterogeneity among patients, by revealing two distinct transcriptome profiles. Patient monocytes sensitive to GM‐CSF have intrinsic activation of the IFN pathway that may facilitate their differentiation towards M1‐MΦ. In contrast, refractory patient monocytes show an overall metabolic de‐activation that may preclude their M1‐MΦ differentiation. IFN signalling has been largely reported as relevant for anti‐tumor responses^64^ and predictive of reduced bone marrow metastasis.^65^ IFN signalling may also exert a protective role by overcoming tumor education in bloodstream as observed in sensitive patient monocytes, confirming previous *in vitro* studies documenting the effect of IFNs on DC or MΦ differentiation.^66^, ^67^ Furthermore, previous studies also reported that circulating patient monocytes present alterations in metabolic pathways in renal cell carcinoma^16^ and metastatic BC patients,^17^ corroborating the fact that tumor cells can alter the monocytes in their periphery. Our study adds another level of complexity by identifying cancer patients with localised primary breast tumors presenting circulating monocytes refractory or sensitive to GM‐CSF differentiation into M1‐MΦs. Importantly, we validate in an independent cohort of BC patients that the sensitive monocyte score is enriched in the type I IFN^high^ group, but the prognostic value of this finding should be further validated in a retrospective cohort.

We also found that LPS‐treated refractory patient monocytes preferentially produce immunosuppressive (TGF‐β1, TGF‐β3 and IL‐10), angiogenic (VEGF) and metastatic‐related (CCL2, CCL4 and CCL5) cytokines. These findings corroborate our previous studies, showing that blood monocytes from primary BC patients have a reduced ability to secrete TNF‐α in response to IFN‐α stimulation^15^ and give rise to TGF‐β‐producing Mo‐DCs.^13^ Moreover, previous studies have also reported that TAM differentiation at distant sites may promote metastasis through the production of chemokines such as CCL2 and CCL5.^68^, ^69^ Thus, the abnormal down‐regulation of cellular metabolic pathways found in refractory monocyte transcriptomes suggests an altered reprogramming capacity preventing their M1‐MΦ differentiation and increasing their suppressive phenotype.

Soluble factors detected in serum previously associated with worse prognosis for BC patients such as TGF‐β,^70^ VEGF and IL‐6^71^ or M‐CSF^72^ may alter monocytes at a distance. Alternatively, as the presence of tumor cells in the bone marrow of BC patients is correlated with worse prognosis,^73^, ^74^ it may suggest that bone marrow invading tumor cells could reprogram monocyte progeny in the bone marrow during their differentiation/maturation.

## Conclusions

Overall, we propose a new mechanism of tumor escape in BC patients. Indeed, complex tumor microenvironmental products appear to act either locally, at the tumor site, or systemically (through the bloodstream and/or bone marrow) to trigger two main monocyte profiles: (1) metabolic impairment, which leads to differentiation and functional biases that favor the tumor growth and (2) IFN enrichment, suggestive of patient protection. These aspects should be considered in the design of personalised immunotherapeutic approaches targeting autologous monocytes/DCs against cancer.

## Methods

### Biological samples

The clinical characteristics of all patients are summarised in Supplementary table 1. Fresh untreated primary breast tumors (BC) (*n* = 93) obtained at the Centre Léon Bérard hospital (CLB) were used for TAM analysis. All of these samples were provided by the tissue bank of CLB (BB‐0033‐00050, CRB‐CLB, Lyon, France, French agreement number: AC‐2013‐1871), after approval from the institutional review board and ethics committee (L‐06‐36 and L‐11‐26) and patient written informed consent, in accordance with the Declaration of Helsinki. Blood samples from 44 patients with untreated primary BC were obtained after signed informed consent from CLB and from Pérola Byington Hospital (São Paulo, Brazil). All samples were anonymously coded in accordance with local ethical guidelines. Healthy donor (HD) blood samples were obtained from the ‘Etablissement Français du Sang’ (Lyon).

### Preparation of tumor supernatants (SNDil) and of total cells from BC

Breast tumors tissues were weighed and dissociated mechanically in an equal volume (w/v) of RPMI‐1640 medium containing antibiotics (penicillin 100 IU mL^−1^ and streptomycin 100 mg L^−1^, Life Technologies, Cailloux‐sur‐Fontaines, France), and supernatants, later referred to in the text as ‘supernatant of dilaceration (SNDil)’, were harvested, filtered (0.22 µm), aliquoted and frozen at −80°C until further use. The mechanically disrupted BC was then enzymatically (Collagenase Ia (1 µg mL^−1^) and DNase‐I (50 kU mL^−1^), Sigma‐Aldrich, Saint‐Quentin‐Fallavier, France) digested for 45 min at 37°C in serum‐free RPMI 1640 medium with antibiotics under agitation. The final cell suspension extracted from BC was washed and resuspended in PBS 2% FCS (Eurobio, Les Ulis, France) plus 0.5 mm EDTA (Sigma‐Aldrich) for FACS analysis and subsequent experiments.

### Isolation of TAMs by FACS and cytospin

Tumor‐associated macrophages were isolated from BC suspensions by cell sorting (FACS Aria‐II, Becton Dickinson) using specific fluorescent antibodies and the following strategy: DAPI^neg^, CD45^+^/CD11b^+^/HLA‐DR^+^/CD14^+^/CD64^+^ CD163^neg/low^ or CD163^high^ (control isotypes were used to define the CD163 gate) as shown in Supplementary figure 1. TAMs were submitted to cytospin centrifugation at 600 rpm for 5 min on glass slides. Cytoplasm and nuclei morphology were then revealed by May–Grünwald/Giemsa staining.

### Immunohistochemistry

A TMA (600 µm core in triplicate) collecting paraffin‐embedded tumors from 238 untreated patients with pathologically confirmed primary BC at the CLB was developed for *in situ* analysis (see Supplementary table 2 for clinical characteristics). CD163 expression was analysed on sections with a mouse IgG1 anti‐human CD163 antibody (clone 10D6, 0.5 µg mL^−1^, reference: NCL‐L‐CD163, Novocastra, Leica Biosystems, Nanterre, France). Optimisation of staining was previously performed by the Pathology Department at the CLB using a monoclonal mouse control isotype (mouse IgG1‐clone MOPC21, reference: PA0996, Novocastra) in distinct human tissues.

### Blood monocyte purification and differentiation into antigen‐presenting cells (APCs) *in vitro*

Peripheral blood mononuclear cells (PBMCs) were obtained by Ficoll density gradient centrifugation (Eurobio). Subsequently, HD monocytes were enriched on 51% Percoll density gradient (GE Healthcare Life Sciences, Buc, France) and CD14^+^ monocytes were purified by negative selection (CD14 isolation kit, Miltenyi Biotec, Paris, France) according to the manufacturer’s instructions (> 95% pure). Isolated monocytes were then differentiated in complete RPMI‐1640 medium (containing antibiotics and 10% heat‐inactivated FCS) for 7 days into the different control APC populations, that is: cultured in medium alone for M0‐MΦ; GM‐CSF (50 ng mL^−1^, Schering‐Plough, Dardilly, France) and IFN‐γ (20 ng mL^−1^, PeproTech, Neuilly‐sur‐Seine, France) for M1‐MΦ; M‐CSF (50 ng mL^−1^, R&D Systems, Lille, France) and IL‐4 (20 ng mL^−1^, Schering‐Plough) for M2‐MΦ; and GM‐CSF (50 ng mL^−1^) and IL‐4 (20 ng mL^−1^) for Mo‐DC. For some experiments, LPS (*Escherichia coli* 0111:B4; 100 ng mL^−1^, Invivogen, Toulouse, France) was added during the last 24 h. For SNDil experiments, purified HD CD14^+^ monocytes were incubated with 25% (v/v) of SNDils for 7 days in complete RPMI medium (SNDil‐MΦ) and LPS was added at day 6 to activate the cells. CD163 relative expression (r‐CD163) was assessed by dividing CD163 MFI from each investigated cell population by CD163 MFI of the internal control (M0‐MΦ) of each experiment. SNDil‐MΦ CD163^high^IL‐10^high^ was considered when r‐CD163 was ≥ 1.6 fold, a value similar to the lowest value of M2‐MΦ; and when IL‐10 concentration was ≥ 415 pg mL^−1^, the highest value of IL‐10 produced by M0‐MΦ.

For experiments using BC patient blood, CD14^+^ monocytes were purified directly from PBMCs by negative selection as above. For some experiments, LPS (100 ng mL^−1^) was added for 24 h.

### Suppression assay

CD4^+^CD45RA^+^ naïve T cells purified by negative selection (Magnisort Human CD4 Naive T cell Enrichment Kit, ThermoFisherScientific, Waltham, Massachusetts, USA) and stained with CellTrace Violet (5 µm, Life Technologies) were cultured in U‐bottom 96‐well plates in the presence of expand beads (anti‐CD3/anti‐CD28 beads, expand beads, Life Technologies). After 30 min of pre‐incubation, LPS‐activated control APCs or SNDil‐MΦ were added in plates for 4 days at a ratio of 1 APC: 2 T cells. CD4^+^ T‐cell proliferation was assessed by the analysis of CTV dilution by flow cytometry. Condition with expand beads alone corresponded to the maximum of proliferation. To assess expansion/suppression of CD4^+^ T lymphocytes, data were normalised by calculating the % of proliferating cells with expand beads + APCs in relation to CD4^+^ T lymphocytes activated by expand beads alone (defined as T‐cell expansion).

### Blocking antibodies

Anti‐IL‐10 (10 µg mL^−1^, clone JES3‐12G8, rat IgG2a, AbD Serotec, Marnes‐la‐Coquette, France), anti‐IL‐10Rα (10 µg mL^−1^, clone 3c.5.2b, mouse IgG1, Schering‐Plough) and anti‐PD‐L1 (20 µg mL^−1^, clone 29E.2A3, mouse IgG2b, Biolegend, Saint‐Cyr‐L’école, France) blocking antibodies and their respective controls were used in the suppression assay and to assess the role of IL‐10 in the modulation of surface markers on *in vitro* generated M2‐MΦ. Anti‐M‐CSF (20 µg mL^−1^, polyclonal rabbit IgG, Genzyme, Lyon, France) and anti‐TGF‐β (10 µg mL^−1^, pan‐specific antibody, polyclonal rabbit IgG, R&D Systems) antibodies and clinical anti‐VEGF antibody (1 µg mL^−1^, human IgG1, Avastin^®^, Roche, Bale, Switzerland) were used in SNDil‐MΦ cultures.

### Cytokine detection in supernatants

IL‐10 and TNF‐α levels were quantified in culture supernatants from control APCs or SNDil‐MΦ by ELISA (Life Technologies and Biolegend, respectively) and analysed with SkanIt software (Thermo Scientific, Dardilly, France). Cytokines and soluble factors were evaluated using multiplex Luminex technology according to the manufacturer’s recommendations as follows: (1) in supernatants of suppression assay with or without anti‐IL10/anti‐PD‐1 blockage with a customised 14‐plex (Millipore, Molsheim, France); (2) in SNDils using 4 different kits: 12plex, 9plex, 8plex and 3plex (Bio‐Rad, Marnes‐la‐Coquette, France); and (3) supernatants from LPS‐activated CD14^+^ blood monocytes from BC patients and HD, with 3 different kits: 23plex, 5plex (Life Technologies) and 3plex (TGF‐β; Bio‐Rad). All assays were analysed on Bio‐Plex 200 (Bio‐Rad) and analysed with Bio‐Plex Manager software.

### Microarray analysis of BC patient monocytes

CD14^+^HLA‐DR^+^ monocytes were FACS‐sorted (> 98% purity) from 8 HDs’ and 8 BC patients’ frozen PBMCs and submitted to total RNA extraction using the microRNeasy kit (Cat. No. 74004; Qiagen, Courtaboeuf, France) according to the manufacturer’s protocol. After RNA integrity number calculation (RIN from samples ≥ 5.7), 500 pg of total RNA (samples ≥ 1.3 ng µL^−1^) was submitted to cDNA synthesis and DNA labelling and hybridisation (GeneChip™ Hybridization, Cat. No. 900720; Applied Biosystems, Thermo Scientific, for subsequent microarray analysis (Clariom™ S Assay human, Cat. No. 902927; Applied Biosystems) using Scanner 3000 7G (Applied Biosystems). Microarray data analysis was performed in R (version 3.5.2, supported by R Foundation for Statistical Computing, Vienna, Austria). Raw probe intensities were quantile normalised and log_2_‐transformed using the Robust Multi‐array Averaging (RMA) method implemented in the oligo R package (v. 1.46.0).^75^ Differentially expressed genes (DEG) between HD and patient monocytes were identified using DESeq2 (v. 1.22.2).^76^ A heat‐map of DEGs was generated using Morpheus from the Broad Institute considering a false discovery rate (FDR) < 0.05 and a log_2_ fold change > 0.58. Hierarchical clustering was based on one minus the Pearson correlation distance. Gene Ontology (GO) analysis was performed with AmiGO web browser^77^ and functional analyses (KEGG and MsigDB hallmark collection) with clusterProfiler package (v. 3.10.1).^78^ Gene set enrichment analyses (GSEA) were represented using BubbleGUM.^79^ Enriched gene sets were considered with a normalised enrichment scores (NES) > 1.5 and FDR < 0.05. Molecular phenotypic signatures were extracted from monocytes of BC patients with the GeneSign tool of BubbleGUM^79^ with the min–max method and a minimal log fold change (LFC) threshold to 1.2. Finally, single‐sample scoring analysis was performed using singscore with default parameters (v 1.2.2) and patients were stratified according to the median of IFN‐alpha response or OXPHOS scores.

### Statistical analysis

For *in situ* analysis of CD163 in IHC studies, the correlation with clinical parameters was assessed using either the chi‐square test or Fisher’s exact test. The impact on BC patient progression‐free survival (PFS) was assessed using the Kaplan–Meier method with SAS software, version 9.2 (SAS Institute, North Caroline, USA). For phenotype and cytokine production of monocytes, MΦ, DCs, SNDil‐MΦ and tumor analysis, we used the one‐way ANOVA test with the Bonferroni post hoc test (GraphPad Prism 6.05, GraphPad Software, San Diego, CA, USA). For cytokine production from LPS‐stimulated blood monocytes, we used an unpaired *t‐*test with the Mann–Whitney *U*‐test comparing refractory, sensitive and HD monocytes. Linear discriminant analysis (LDA) using cytokine production was applied to predict patient monocyte classification into refractory versus sensitive groups (XLStat software, Addinsoft, Paris, France).

## Conflict of interest

The authors declare no conflict of interest.

## Author contributions

RNR performed the experiments, analysed the data, wrote the paper, and designed and interpreted the results with CC, CMC and NBV. CR performed the experiments, analysed the data and participated in the interpretation and discussion of reported results. IT, OT, CHR, HGG, FL and AC participated in patients’ sample selection for fresh tumor analysis and IHC analysis of the retrospective cohort of primary BC patients. EL, SC and AC performed the statistical analysis for the prognostic impact of CD163 expression. MH helped in the experiments of cytokine measurement. MH, MA and WR performed bioinformatics analyses of microarray results from patients’ monocytes data. EP and JAMB helped in the discussion and in data interpretation and reviewed the manuscript. CC, CMC and NBV supervised the study and reviewed the manuscript.

## Ethics approval and consent to participate

All patients have consent and approved the use of their biological tissue. This study was approved by the ethics committee of the Centre Leon Bérard Hospital (Lyon, France) and from Pérola Byington Hospital (São Paulo, Brazil).

## References

[1] Caux C , Ramos RN , Prendergast GC *et al* A milestone review on how macrophages affect tumor growth. Cancer Res 2016; 76: 6439–6442. 28148676 10.1158/0008-5472.CAN-16-2631

[2] Qian BZ , Pollard JW . Macrophage diversity enhances tumor progression and metastasis. Cell 2010; 141: 39–51. 20371344 10.1016/j.cell.2010.03.014 PMC4994190

[3] Pollard JW . Trophic macrophages in development and disease. Nat Rev Immunol 2009; 9: 259–270. 19282852 10.1038/nri2528 PMC3648866

[4] Campbell MJ , Tonlaar NY , Garwood ER *et al* Proliferating macrophages associated with high grade, hormone receptor negative breast cancer and poor clinical outcome. Breast Cancer Res Treat 2011; 128: 703–711. 20842526 10.1007/s10549-010-1154-y PMC4657137

[5] Lacey DC , Achuthan A , Fleetwood AJ *et al* Defining GM‐CSF‐ and macrophage‐CSF‐dependent macrophage responses by *in vitro* models. J Immunol 2012; 188: 5752–5765. 22547697 10.4049/jimmunol.1103426

[6] Jaguin M , Houlbert N , Fardel O *et al* Polarization profiles of human M‐CSF‐generated macrophages and comparison of M1‐markers in classically activated macrophages from GM‐CSF and M‐CSF origin. Cell Immunol 2013; 281: 51–61. 23454681 10.1016/j.cellimm.2013.01.010

[7] Kuang DM , Zhao Q , Peng C *et al* Activated monocytes in peritumoral stroma of hepatocellular carcinoma foster immune privilege and disease progression through PD‐L1. J Exp Med 2009; 206: 1327–1337. 19451266 10.1084/jem.20082173 PMC2715058

[8] Pollard JW . Tumour‐educated macrophages promote tumour progression and metastasis. Nat Rev Cancer 2004; 4: 71–78. 14708027 10.1038/nrc1256

[9] Azizi E , Carr AJ , Plitas G *et al* Single‐cell map of diverse immune phenotypes in the breast tumor microenvironment. Cell 2018; 174: 1293–1308. 29961579 10.1016/j.cell.2018.05.060 PMC6348010

[10] Chevrier S , Levine JH , Zanotelli VRT *et al* An immune atlas of clear cell renal cell carcinoma. Cell 2017; 169: 736–749.e18. 28475899 10.1016/j.cell.2017.04.016 PMC5422211

[11] Lavin Y , Kobayashi S , Leader A *et al* Innate immune landscape in early lung adenocarcinoma by paired single‐cell analyses. Cell 2017; 169: 750–765. 28475900 10.1016/j.cell.2017.04.014 PMC5737939

[12] McAllister SS , Weinberg RA . The tumour‐induced systemic environment as a critical regulator of cancer progression and metastasis. Nat Cell Biol 2014; 16: 717–727. 25082194 10.1038/ncb3015 PMC6220424

[13] Ramos RN , Chin LS , Dos Santos AP *et al* Monocyte‐derived dendritic cells from breast cancer patients are biased to induce CD4^+^CD25^+^Foxp3^+^ regulatory T cells. J Leukoc Biol 2012; 92: 673–682. 22636320 10.1189/jlb.0112048

[14] Ramos RN , de Moraes CJ , Zelante B *et al* What are the molecules involved in regulatory T‐cells induction by dendritic cells in cancer? Clin Dev Immunol 2013; 2013: 806025. 23762097 10.1155/2013/806025 PMC3674660

[15] Verronese E , Delgado A , Valladeau‐Guilemond J *et al* Immune cell dysfunctions in breast cancer patients detected through whole blood multi‐parametric flow cytometry assay. Oncoimmunology 2016; 5: e1100791. 27141361 10.1080/2162402X.2015.1100791 PMC4839376

[16] Chittezhath M , Dhillon MK , Lim JY *et al* Molecular profiling reveals a tumor‐promoting phenotype of monocytes and macrophages in human cancer progression. Immunity 2014; 41: 815–829. 25453823 10.1016/j.immuni.2014.09.014

[17] Bergenfelz C , Larsson AM , von Stedingk K *et al* Systemic monocytic‐MDSCs are generated from monocytes and correlate with disease progression in breast cancer patients. PLoS One 2015; 10: e0127028. 25992611 10.1371/journal.pone.0127028 PMC4439153

[18] Cassetta L , Fragkogianni S , Sims AH *et al* Human Tumor‐Associated Macrophage and monocyte transcriptional landscapes reveal cancer‐specific reprogramming, biomarkers, and therapeutic targets. Cancer Cell 2019; 35: 588–602.e10. 30930117 10.1016/j.ccell.2019.02.009 PMC6472943

[19] Foulds GA , Vadakekolathu J , Abdel‐Fatah TMA *et al* Immune‐phenotyping and transcriptomic profiling of peripheral blood mononuclear cells from patients with breast cancer: identification of a 3 gene signature which predicts relapse of triple negative breast cancer. Front Immunol 2018; 9: 2028. 30254632 10.3389/fimmu.2018.02028 PMC6141692

[20] Caso R , Silvera R , Carrio R *et al* Blood monocytes from mammary tumor‐bearing mice: early targets of tumor‐induced immune suppression? Int J Oncol 2010; 37: 891–900. 20811711 10.3892/ijo_00000740

[21] Torroella‐Kouri M , Rodriguez D , Caso R . Alterations in macrophages and monocytes from tumor‐bearing mice: evidence of local and systemic immune impairment. Immunol Res 2013; 57: 86–98. 24203436 10.1007/s12026-013-8438-3

[22] Stone SC , Rossetti RA , Bolpetti A *et al* HPV16‐associated tumors control myeloid cell homeostasis in lymphoid organs, generating a suppressor environment for T cells. J Leukoc Biol 2014; 96: 619–631. 24970861 10.1189/jlb.3A0513-282R

[23] Goudot C , Coillard A , Villani A‐C *et al* Aryl hydrocarbon receptor controls monocyte differentiation into dendritic cells versus macrophages. Immunity 2017; 47: 582–596.e6. 28930664 10.1016/j.immuni.2017.08.016

[24] Hacker C , Kirsch RD , Ju X‐S *et al* Transcriptional profiling identifies Id2 function in dendritic cell development. Nat Immunol 2003; 4: 380–386. 12598895 10.1038/ni903

[25] Pinho MP , Migliori IK , Flatow EA *et al* Dendritic cell membrane CD83 enhances immune responses by boosting intracellular calcium release in T lymphocytes. J Leukoc Biol 2014; 95: 755–762. 24436459 10.1189/jlb.0413239

[26] Ben‐Sasson SZ , Hu‐Li J , Quiel J *et al* IL‐1 acts directly on CD4 T cells to enhance their antigen‐driven expansion and differentiation. Proc Natl Acad Sci USA 2009; 106: 7119–7124. 19359475 10.1073/pnas.0902745106 PMC2678417

[27] De Maria R , Cifone MG , Trotta R *et al* Triggering of human monocyte activation through CD69, a member of the natural killer cell gene complex family of signal transducing receptors. J Exp Med 1994; 180: 1999–2004. 7964477 10.1084/jem.180.5.1999 PMC2191715

[28] de Buzzo CL , Medina T , Branco LM *et al* Epigenetic regulation of nitric oxide synthase 2, inducible (Nos2) by NLRC4 inflammasomes involves PARP1 cleavage. Sci Rep 2017; 7: 41686. 28150715 10.1038/srep41686 PMC5288713

[29] Wagner J , Rapsomaniki MA , Chevrier S , et al. A single-cell atlas of the tumor and immune ecosystem of human breast cancer. Cell 2019; 177: 1330–1345.e18. 30982598 10.1016/j.cell.2019.03.005 PMC6526772

[30] Li H , van der Leun AM , Yofe I *et al* Dysfunctional CD8 T cells form a proliferative, dynamically regulated compartment within human melanoma. Cell 2019; 176: 775–789.e18. 30595452 10.1016/j.cell.2018.11.043 PMC7253294

[31] Zilionis R , Engblom C , Pfirschke C *et al* Single‐cell transcriptomics of human and mouse lung cancers reveals conserved myeloid populations across individuals and species. Immunity 2019; 50: 1317–1334.e10. 30979687 10.1016/j.immuni.2019.03.009 PMC6620049

[32] Cassetta L , Pollard JW . Targeting macrophages: therapeutic approaches in cancer. Nat Rev Drug Discov 2018; 17: 887–904. 30361552 10.1038/nrd.2018.169

[33] Kim IS , Gao Y , Welte T *et al* Immuno‐subtyping of breast cancer reveals distinct myeloid cell profiles and immunotherapy resistance mechanisms. Nat Cell Biol 2019; 21: 1113–1126. 31451770 10.1038/s41556-019-0373-7 PMC6726554

[34] Gubin MM , Esaulova E , Ward JP *et al* High‐dimensional analysis delineates myeloid and lymphoid compartment remodeling during successful immune‐checkpoint cancer therapy. Cell 2018; 175: 1014–1030.e19. 30343900 10.1016/j.cell.2018.09.030 PMC6501221

[35] Medrek C , Ponten F , Jirstrom K *et al* The presence of tumor associated macrophages in tumor stroma as a prognostic marker for breast cancer patients. BMC Cancer 2012; 12: 306. 22824040 10.1186/1471-2407-12-306 PMC3414782

[36] Pelekanou V , Villarroel‐Espindola F , Schalper KA *et al* CD68, CD163, and matrix metalloproteinase 9 (MMP‐9) co‐localization in breast tumor microenvironment predicts survival differently in ER‐positive and ‐negative cancers. Breast Cancer Res 2018; 20: 154. 30558648 10.1186/s13058-018-1076-x PMC6298021

[37] Kruger JM , Wemmert C , Sternberger L *et al* Combat or surveillance? Evaluation of the heterogeneous inflammatory breast cancer microenvironment. J Pathol 2013; 229: 569–578. 23192518 10.1002/path.4150

[38] Sousa S , Brion R , Lintunen M *et al* Human breast cancer cells educate macrophages toward the M2 activation status. Breast Cancer Res 2015; 17: 101. 26243145 10.1186/s13058-015-0621-0 PMC4531540

[39] Jeong H , Hwang I , Kang SH *et al* Tumor‐Associated Macrophages as potential prognostic biomarkers of invasive breast cancer. J Breast Cancer 2019; 22: 38. 30941232 10.4048/jbc.2019.22.e5 PMC6438840

[40] Bottai G , Raschioni C , Székely B *et al* AXL‐associated tumor inflammation as a poor prognostic signature in chemotherapy‐treated triple‐negative breast cancer patients. NPJ Breast Cancer 2016; 2: 16033. 28721387 10.1038/npjbcancer.2016.33 PMC5515347

[41] Nicolau M , Levine AJ , Carlsson G . Topology based data analysis identifies a subgroup of breast cancers with a unique mutational profile and excellent survival. Proc Natl Acad Sci USA 2011; 108: 7265–7270. 21482760 10.1073/pnas.1102826108 PMC3084136

[42] Volodko N , Gutor T , Petronchak O *et al* Low infiltration of tumor‐associated macrophages in high c‐Myb‐expressing breast tumors. Sci Rep 2019; 9: 11634. 31406165 10.1038/s41598-019-48051-1 PMC6690941

[43] Honkanen TJ , Tikkanen A , Karihtala P *et al* Prognostic and predictive role of tumour‐associated macrophages in HER2 positive breast cancer. Sci Rep 2019; 9: 10961. 31358801 10.1038/s41598-019-47375-2 PMC6662906

[44] Hoves S , Ooi C‐H , Wolter C *et al* Rapid activation of tumor‐associated macrophages boosts preexisting tumor immunity. J Exp Med 2018; 215: 859–876. 29436396 10.1084/jem.20171440 PMC5839760

[45] Etzerodt A , Tsalkitzi K , Maniecki M *et al* Specific targeting of CD163^+^ TAMs mobilizes inflammatory monocytes and promotes T cell–mediated tumor regression. J Exp Med 2019; 216: 2394–2411. 31375534 10.1084/jem.20182124 PMC6781002

[46] DeNardo DG , Brennan DJ , Rexhepaj E *et al* Leukocyte complexity predicts breast cancer survival and functionally regulates response to chemotherapy. Cancer Discov 2011; 1: 54–67. 22039576 10.1158/2159-8274.CD-10-0028 PMC3203524

[47] Doedens AL , Stockmann C , Rubinstein MP *et al* Macrophage expression of hypoxia‐inducible factor‐1 α suppresses T‐cell function and promotes tumor progression. Cancer Res 2010; 70: 7465–7475. 20841473 10.1158/0008-5472.CAN-10-1439 PMC2948598

[48] Movahedi K , Laoui D , Gysemans C *et al* Different tumor microenvironments contain functionally distinct subsets of macrophages derived from Ly6C(high) monocytes. Cancer Res 2010; 70: 5728–5739. 20570887 10.1158/0008-5472.CAN-09-4672

[49] Ruffell B , Chang‐Strachan D , Chan V *et al* Macrophage IL‐10 blocks CD8^+^ T cell‐dependent responses to chemotherapy by suppressing IL‐12 expression in intratumoral dendritic cells. Cancer Cell 2014; 26: 623–637. 25446896 10.1016/j.ccell.2014.09.006 PMC4254570

[50] Uzzan B , Nicolas P , Cucherat M *et al* Microvessel density as a prognostic factor in women with breast cancer: a systematic review of the literature and meta‐analysis. Cancer Res 2004; 64: 2941–2955. 15126324 10.1158/0008-5472.can-03-1957

[51] Bolat F , Kayaselcuk F , Nursal TZ *et al* Microvessel density, VEGF expression, and tumor‐associated macrophages in breast tumors: correlations with prognostic parameters. J Exp Clin Cancer Res 2006; 25: 365–372. 17167977

[52] Biswas SK , Gangi L , Paul S *et al* A distinct and unique transcriptional program expressed by tumor‐associated macrophages (defective NF‐κB and enhanced IRF‐3/STAT1 activation). Blood 2006; 107: 2112–2122. 16269622 10.1182/blood-2005-01-0428

[53] Belai EB , de Oliveira CE , Gasparoto TH *et al* PD‐1 blockage delays murine squamous cell carcinoma development. Carcinogenesis 2014; 35: 424–431. 24031027 10.1093/carcin/bgt305

[54] Menetrier‐Caux C , Montmain G , Dieu MC *et al* Inhibition of the differentiation of dendritic cells from CD34^+^ progenitors by tumor cells: role of interleukin‐6 and macrophage colony‐stimulating factor. Blood 1998; 92: 4778–4791. 9845545

[55] Menetrier‐Caux C , Thomachot MC , Alberti L *et al* IL‐4 prevents the blockade of dendritic cell differentiation induced by tumor cells. Cancer Res 2001; 61: 3096–3104. 11306493

[56] Ma GF , Miao Q , Zeng XQ *et al* Transforming growth factor‐β1 and ‐β2 in gastric precancer and cancer and roles in tumor‐cell interactions with peripheral blood mononuclear cells *in vitro* . PLoS One 2013; 8: e54249. 23342108 10.1371/journal.pone.0054249 PMC3544811

[57] Shabo I , Stal O , Olsson H *et al* Breast cancer expression of CD163, a macrophage scavenger receptor, is related to early distant recurrence and reduced patient survival. Int J Cancer 2008; 123: 780–786. 18506688 10.1002/ijc.23527

[58] Arwert EN , Harney AS , Entenberg D *et al* A unidirectional transition from migratory to perivascular macrophage is required for tumor cell intravasation. Cell Rep 2018; 23: 1239–1248. 29719241 10.1016/j.celrep.2018.04.007 PMC5946803

[59] Sisirak V , Vey N , Goutagny N *et al* Breast cancer‐derived transforming growth factor‐β and tumor necrosis factor‐α compromise interferon‐α production by tumor‐associated plasmacytoid dendritic cells. Int J Cancer 2013; 133: 771–778. 23389942 10.1002/ijc.28072

[60] Nie Y , Huang H , Guo M *et al* Breast phyllodes tumors recruit and repolarize tumor‐associated macrophages via secreting CCL5 to promote malignant progression, which can be inhibited by CCR5 inhibition therapy. Clin Cancer Res 2019; 25: 3873–3886. 30890553 10.1158/1078-0432.CCR-18-3421

[61] Park JE , Dutta B , Tse SW *et al* Hypoxia‐induced tumor exosomes promote M2‐like macrophage polarization of infiltrating myeloid cells and microRNA‐mediated metabolic shift. Oncogene 2019; 38: 5158–5173. 30872795 10.1038/s41388-019-0782-x

[62] Clavijo‐Salomon MA , Ramos RN , Crippa A *et al* Monocyte‐derived dendritic cells reflect the immune functional status of a chromophobe renal cell carcinoma patient: could it be a general phenomenon? Cancer Immunol Immunother 2015; 64: 161–171. 25314913 10.1007/s00262-014-1625-9 PMC11029287

[63] Neves AR , Ensina LF , Anselmo LB *et al* Dendritic cells derived from metastatic cancer patients vaccinated with allogeneic dendritic cell‐autologous tumor cell hybrids express more CD86 and induce higher levels of interferon‐γ in mixed lymphocyte reactions. Cancer Immunol Immunother 2005; 54: 61–66. 15693140 10.1007/s00262-004-0550-8 PMC11034268

[64] Parker BS , Rautela J , Hertzog PJ . Antitumour actions of interferons: implications for cancer therapy. Nat Rev Cancer 2016; 16: 131–144. 26911188 10.1038/nrc.2016.14

[65] Bidwell BN , Slaney CY , Withana NP *et al* Silencing of Irf7 pathways in breast cancer cells promotes bone metastasis through immune escape. Nat Med 2012; 18: 1224–1231. 22820642 10.1038/nm.2830

[66] Mohty M , Vialle‐Castellano A , Nunes JA *et al* IFN‐α skews monocyte differentiation into Toll‐like receptor 7‐expressing dendritic cells with potent functional activities. J Immunol 2003; 171: 3385–3393. 14500632 10.4049/jimmunol.171.7.3385

[67] Delneste Y , Charbonnier P , Herbault N *et al* Interferon‐γ switches monocyte differentiation from dendritic cells to macrophages. Blood 2003; 101: 143–150. 12393446 10.1182/blood-2002-04-1164

[68] Frankenberger C , Rabe D , Bainer R *et al* Metastasis suppressors regulate the tumor microenvironment by blocking recruitment of prometastatic tumor‐associated macrophages. Cancer Res 2015; 75: 4063–4073. 26238785 10.1158/0008-5472.CAN-14-3394 PMC4592465

[69] Svensson S , Abrahamsson A , Rodriguez GV *et al* CCL2 and CCL5 are novel therapeutic targets for estrogen‐dependent breast cancer. Clin Cancer Res 2015; 21: 3794–3805. 25901081 10.1158/1078-0432.CCR-15-0204

[70] Ivanovic V , Todorovic‐Rakovic N , Demajo M *et al* Elevated plasma levels of transforming growth factor‐β1 (TGF‐β1) in patients with advanced breast cancer: association with disease progression. Eur J Cancer 2003; 39: 454–461. 12751375 10.1016/s0959-8049(02)00502-6

[71] Bachelot T , Ray‐Coquard I , Menetrier‐Caux C *et al* Prognostic value of serum levels of interleukin 6 and of serum and plasma levels of vascular endothelial growth factor in hormone‐refractory metastatic breast cancer patients. Br J Cancer 2003; 88: 1721–1726. 12771987 10.1038/sj.bjc.6600956 PMC2377148

[72] Aharinejad S , Salama M , Paulus P *et al* Elevated CSF1 serum concentration predicts poor overall survival in women with early breast cancer. Endocr Relat Cancer 2013; 20: 777–783. 24016870 10.1530/ERC-13-0198

[73] Braun S , Pantel K , Muller P *et al* Cytokeratin‐positive cells in the bone marrow and survival of patients with stage I, II, or III breast cancer. N Engl J Med 2000; 342: 525–533. 10684910 10.1056/NEJM200002243420801

[74] Braun S , Vogl FD , Naume B *et al* A pooled analysis of bone marrow micrometastasis in breast cancer. N Engl J Med 2005; 353: 793–802. 16120859 10.1056/NEJMoa050434

[75] Carvalho BS , Irizarry RA . A framework for oligonucleotide microarray preprocessing. Bioinformatics 2010; 26: 2363–2367. 20688976 10.1093/bioinformatics/btq431 PMC2944196

[76] Love MI , Huber W , Anders S . Moderated estimation of fold change and dispersion for RNA‐seq data with DESeq2. Genome Biol 2014; 15: 550. 25516281 10.1186/s13059-014-0550-8 PMC4302049

[77] Carbon S , Ireland A , Mungall CJ *et al* AmiGO: online access to ontology and annotation data. Bioinformatics 2009; 25: 288–289. 19033274 10.1093/bioinformatics/btn615 PMC2639003

[78] Yu G , Wang L‐G , Han Y *et al* clusterProfiler: an R package for comparing biological themes among gene clusters. OMICS 2012; 16: 284–287. 22455463 10.1089/omi.2011.0118 PMC3339379

[79] Spinelli L , Carpentier S , Montañana Sanchis F *et al* BubbleGUM: automatic extraction of phenotype molecular signatures and comprehensive visualization of multiple Gene Set Enrichment Analyses. BMC Genom 2015; 16: 814. 10.1186/s12864-015-2012-4 PMC4617899 26481321

