# Supplementary figures and images for: CD163+ tumor‐associated macrophage accumulation in breast cancer patients reflects both local differentiation signals and systemic skewing of monocytes
Source: Clin Transl Immunology. 2020 Feb 13;9(2):e1108. doi: 10.1002/cti2.1108 (PMC7017151; doi:10.1002/cti2.1108)

# Suppl. Figure 1

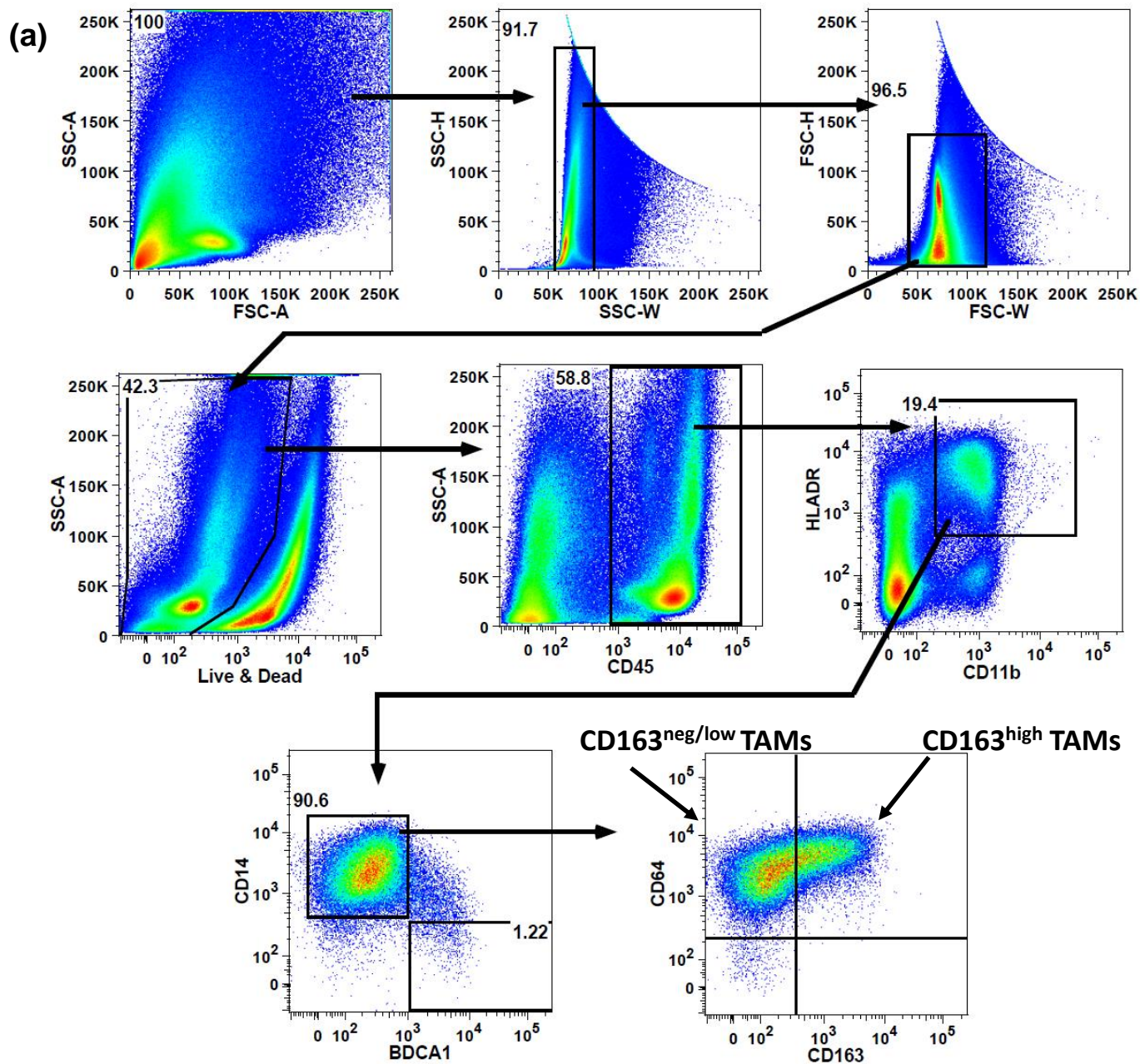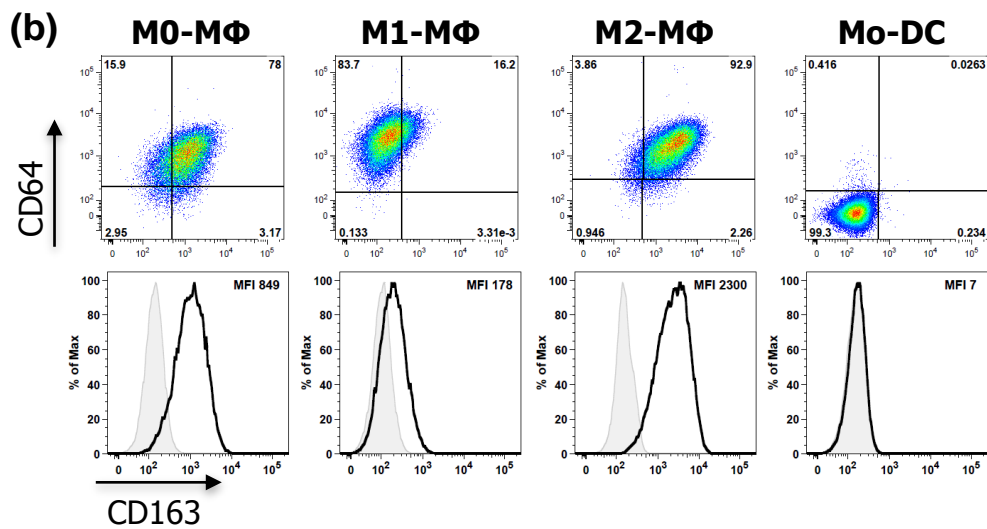

Supplement: Supplementary file 1 [file CTI2-9-e1108-s001.pdf]

# Suppl. Figure 3

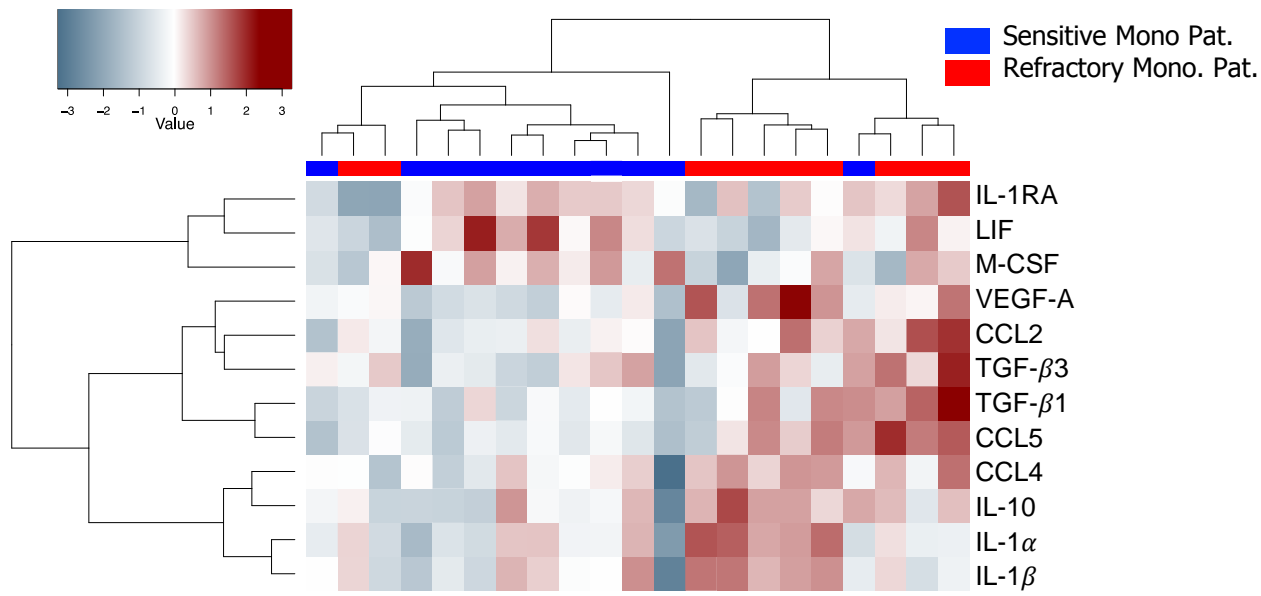

Supplement: Supplementary file 3 [file CTI2-9-e1108-s003.pdf]
