# Supplementary material for: CD163+ tumor‐associated macrophage accumulation in breast cancer patients reflects both local differentiation signals and systemic skewing of monocytes
Source: Clin Transl Immunology. 2020 Feb 13;9(2):e1108. doi: 10.1002/cti2.1108 (PMC7017151; doi:10.1002/cti2.1108)
Supplement: Supplementary file 2 [file CTI2-9-e1108-s002.pdf]

Suppl. Figure 2

(a)

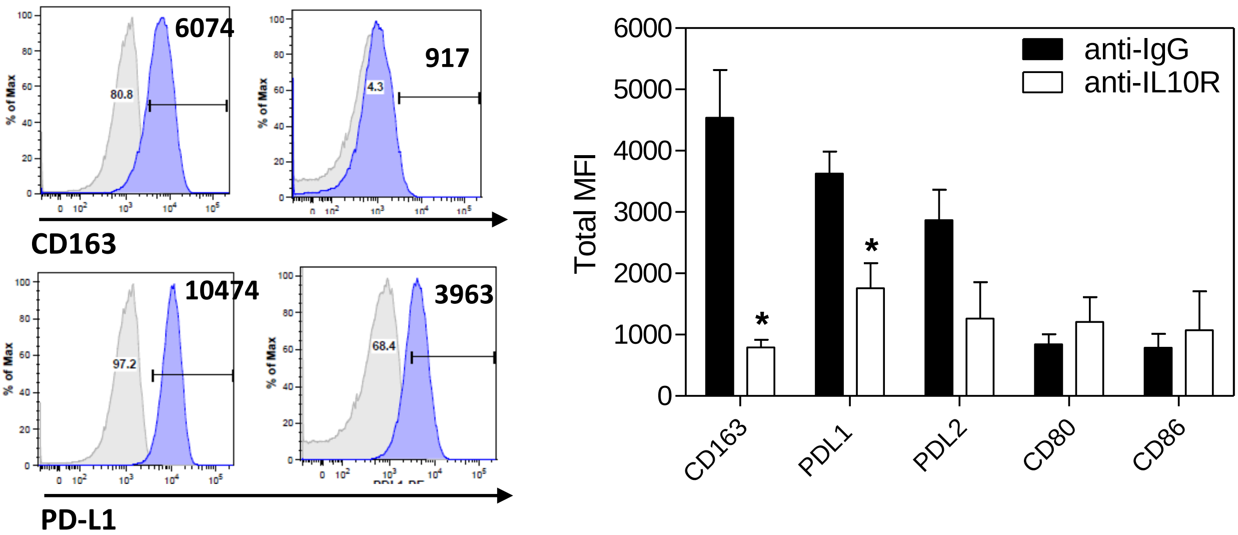

(b)

SNDil content	SNDil-MΦ phenotype	
Cytokines (pg/ml)	$CD163^{low}$ $IL\text{-}10^{low}$	$CD163^{high}$ $IL\text{-}10^{high}$
CCL2	209 ± 163	374± 212
M-CSF	93.5 ± 47	367 ± 144
TGF-β1	796 ± 190	1014 ± 187
TGF-β3	23 ± 4.1	35 ± 6.2
VEGF	1,791 ± 966.7	4,864 ± 2,885
