# Supplementary material for: CD163+ tumor‐associated macrophage accumulation in breast cancer patients reflects both local differentiation signals and systemic skewing of monocytes
Source: Clin Transl Immunology. 2020 Feb 13;9(2):e1108. doi: 10.1002/cti2.1108 (PMC7017151; doi:10.1002/cti2.1108)
Supplement: Supplementary file 4 [file CTI2-9-e1108-s004.pdf]

Supplementary Table 1 - Clinical characteristics of BC patients used for Flow Cytometry analysis

		n	**ratio <1** CD163high/163low	**ratio ≥1** CD163high/163low
age	<50	27 (29%)	19 (70,3%)	8 (29,6%)
	≥50	66 (71%)	47 (71,2%)	19 (28,8%)
menopause	NA	3 (3,2%)		
	no	24 (25,8%)	16 (66,6%)	8 (33,3%)
	yes	66 (70,96%)	47 (71,2%)	19 (28,8%)
histology	ductal	66 (71%)	49 (74,4%)	17 (25,75%)
	lobular	20 (21,5%)	14 (70%)	6 (30%)
	mixte	3 (3,2%)	2 (66,6%)	1 (33,3%)
	others	4 (4,3%)	1 (25%)	3 (75%)
SBR	1	2 (2,1%)	2 (100%)	0
	2	42 (45,1%)	29 (69%)	13 (30,9%)
	3	49 (52,7%)	35 (71%)	14 (28,5%)
N+	no	53 (57%)	36 (67,9%)	17 (32%)
	yes	37 (43%)	29 (78,3%)	8 (21,6%)
Neoadj treatment	no	82 (88,2%)	60 (73%)	22 (26,8%)
	yes	11 (11,8%)	6 (54%)	5 (45%)
ER+	no	24 (25,8%)	18 (75%)	6 (25%)
	yes	69 (74,2%)	48 (69,5%)	21 (30,4%)
PgR+	no	31 (33,3%)	23 (74,1%)	8 (25,8%)
	yes	62 (66,6%)	43 (69,3%)	19 (30,6%)
Her2	no	77 (82,8%)	53 (68,8%)	24 (31,1%)
	yes	16 (17,2%)	13 (81,25%)	3 (18,75%)
TNBT	no	76 (81,7%)	53 (69%)	23 (30%)
	yes	17 (18,3%)	13 (76%)	4 (23%)
Relapse	no	71 (76,3%)	48 (67%)	23 (32%)
	yes	22 (23,7%)	18 (81%)	4 (18%)
