# Supplementary material for: CD163+ tumor‐associated macrophage accumulation in breast cancer patients reflects both local differentiation signals and systemic skewing of monocytes
Source: Clin Transl Immunology. 2020 Feb 13;9(2):e1108. doi: 10.1002/cti2.1108 (PMC7017151; doi:10.1002/cti2.1108)
Supplement: Supplementary file 5 [file CTI2-9-e1108-s005.pdf]

Supplementary Table 2 : Clinical characteristics of BC patients enrolled in the IHC study

	N (%)	CD163		
		0	1-2	p value
N	238	109	129	
**Age (Years)**				0,143
median	57(27,3-87,4)	57 (27,3 87,4)	56.8 (30,4 - 82,7)	
<50	77 (32,4)	30 (27,5)	47 (37,1)	
>50	161 (67,6)	79 (72,5)	82 (62,9)	
**Tumor size (mm)**				0,145
<30	152 (63,9)	75 (68,8)	77 (59,7)	
≥ 30	86 (36,1)	34 (31,2)	52 (40,3)	
**Menopause**				0,071
Unknown	3	3	0	
No	81 (34,5)	30 (28,3)	51 (40,5)	
Yes	154 (65,5)	76 (71,7)	78 (59,5)	
**SBR grade**				**<0,001**
1	38 (16)	30 (27,5)	8 (6,9)	
2	99 (41,6)	53 (46,8)	46 (37,9)	
3	101 (42,4)	26 (23,9)	75 (55,2)	
**Lymph node involvement**				**0,003**
unknown	1	0	1	
**no**	87 (36,7)	51 (46,8)	36 (27)	
yes	150 (63,3)	58 (53,2)	92 (73)	
**Lymphatic emboles**				**0,002**
No	118 (49,6)	66 (60,6)	52 (40,3)	
Yes	120 (50,4)	43 (39,4)	77 (59,7)	
**Molecular subtypes**				**<0,001**
Unknown	11	6	5	
Luminal A	116 (51,1)	74 (71,8)	42 (36,9)	
Luminal B	85 (7,4)	24 (23,3)	61 (50,5)	
Her2/neu amplified	6 (2,6)	2 (1,9)	4 (2,7)	
Triple Negative	20 (8,8)	3 (2,9)	17 (9,9)	
